# Nitrous Oxide Gas an Anæsthetic

**Published:** 1864-08

**Authors:** G. Q. Colton


### NITROUS OXIDE GAS AN ANÆSTHETIC.

*How to use it successfully.*

BY G. Q. COLTON.

It is now about fifteen months since I introduced (or re-introduced) the nitrous oxide as an anæsthetic to the dental profession. During this time I have administered it daily for the extraction of teeth, the number of patients usually varying from six to twelve per day. I have never had the slightest ill effects to attend the operation; and whether we have extracted one or twenty teeth and stumps at a sitting, no patient has desired even to lie down after the teeth were out. Yet experience has taught me much in the anæsthetic use of the gas. I am satisfied that the failures and ill effects attending it, of which complaint is sometimes made, are owing to a failure to comply with certain important rules in making and administering it. These rules I propose briefly to state:

*First*—The gas must be pure; and the best way to test it, is for the dentist to take a few inhalations. If it is pleasant and grateful to the lungs, it is all right.

*Second*—It should be used within forty-eight hours after making. The more fresh the better, after being properly purified.

*Third*—It should be administered from a six gallon bag full. While a four gallon bag will answer for children, I have found an eight gallon necessary in a few rare instances. Too small a bag, and too small an opening in the mouth piece, have been the causes of more failures than all other things put together. The larger the bag, the better the patient will feel after the operation is over.

*Fourth*—The opening in the mouth-piece should not be less than a full half inch.

*Fifth*—Have the patient thoroughly exhaust the lungs before closing the lips on the mouth piece. Keep the nose and lips closed so as not to dilute the gas with air.

*Sixth*—Explain to the patient before commencing the sensations he will experience. "You will think at first that you are breathing only common air,—very soon you will experience a whizzing in the head, and then you will fear that I may suppose you are asleep. You may dismiss all such fears. *I* shall know when you are asleep." This will give confidence to the patient, especially after he commences breathing, and prevents his becoming frightened. After the operation, and as the patient is waking, say to him, "*all right, all right, your teeth are out!*" This arrests his attention, and reminds him of where he is, and what has been done. It allays all fears.

If the bag is too small, the oxygen will be absorbed before anæsthesia is induced, and very labored breathing, gasping, &c., will result. This gasping is for more oxygen, and is never experienced when the gas is administered in sufficient quantity. The same unpleasant results would follow the inhalation of a small bag of common air, only the symptoms would be manifested sooner, because the air contains less oxygen than the gas. Another effect of inhaling from too small a dose, is that the patient will often experience head ache and general weakness after the operation is over. All these bad effects are avoided by administering from a six or eight gallon bag, with a free opening of half an inch. It is true that anæsthesia can be induced, in many cases, with a small dose of nitrous oxide, but the effect is partly the result of *partial suffocation;* and hence the unpleasant effects above alluded too.

A dentist writing to the *Dental Cosmos*, asserts that *nitrous oxide alone* will not induce anæsthesia, but that the result is owing to the narcotizing effects of the carbonic acid gas, which is formed by breathing out and into the bag. He cites as evidence the labored breathing to which I have referred. It is a sufficient answer to this writer, to state, that if a patient breathes from *a hundred gallon bag* (where, of course, he will breathe next to no carbonic acid), anæsthesia will be be induced in a much less space of time than if he breathes from a six gallon bag. Any dentist can test the truth of this. No doubt the writer in the *Cosmos* has administered, or seen administered, too small doses, and the inferences he draws from the unpleasant symptoms were legitimate. Let him follow the above rules, and he will change his opinion respecting the anæsthetic powers of nitrous oxide. If I can administer the gas to two thousand patients, as I have done, without a single failure to produce anæsthesia, and without the slightest ill effects to attend or follow the operation, surely *any* dentist can do it, who will adapt the same plan in administering it.

It is a remarkable fact that although thousands of dentists have used the gas for a year past, *not a single death has occurred while the patient was breathing the gas, or while in the anæsthetic state!* Only one death has occurred soon after the anæsthetic effects had passed off, and this was fully accounted for from other causes. Is not this ample proof that the gas is vastly superior to ether and chloroform in point of safety? No candid person can question it.

As the anæsthetic use of the gas has been thoroughly endorsed by such eminent chemists as PROF. R. OGDEN DOREMUS, PROF. P. H. VANDER WEYDE, and others, and as I have fully tested its safety and efficiency, by a year's practice, it does not become *a dentist* who knows little of chemistry to condemn it upon imperfect and impartial trials, and certainly not till he has tested it by something like the above rules. Prof. Vander Weyde closes a letter upon the subject with the following sentences:

"The difference between the nitrous oxide and ether and chloroform, is, that the first being a supporter of combustion and respiration, stimulates the nervons system and produces an increase of vitality, while the two others, ether and chloroform, being non-supporters of combustion and respiration, depress the nervous system and brings vitality below the standard, though with the same final result, *perfect unconsciousness;* the difference only is that the unconsciousness produced by the increase of vital action with the nitrous oxide is harmless, and the same result produced by the depression of vital action by ether and chloroform is injurious, and may prove fatal."

